# Phase-Separated Coacervates of Low-Molecular-Weight Compounds for Cytosolic Delivery and Disease Treatment

**DOI:** 10.1021/jacsau.5c01164

**Published:** 2025-10-28

**Authors:** Yishu Bao, Jiang Xia

**Affiliations:** a Department of Chemistry, 26451 The Chinese University of Hong Kong, Shatin, Hong Kong SAR 99999, China; b AoE Centre for Plant Vacuole Biology and Biotechnology, 26451 The Chinese University of Hong Kong, Shatin 99999, Hong Kong, China

**Keywords:** LMWC-based coacervates, internalization mechanisms, intracellular delivery, stimuli-responsive, cell surface engineering, therapeutics

## Abstract

Liquid–liquid phase separation (LLPS)-driven coacervate droplets, formed by the self-assembly of phase-separating molecules, have emerged as a new platform for the intracellular delivery of macromolecular therapeutics such as antibodies, plasmids, and mRNA. Their appeal lies in their high loading capacity, low cytotoxicity, and high cellular uptake efficiency. Beyond traditional polymer and protein systems, recent advances have demonstrated that low-molecular-weight compounds, including peptides and small molecules, can also form functional coacervates. In this perspective, we will discuss and elucidate the possible mechanisms underlying coacervate cellular uptake and highlight their applications in macromolecular delivery and disease therapy. We also provide our perspective on future research directions and translational opportunities. By critically evaluating these aspects, we aim to bridge fundamental insights with translational potential while providing a promising strategy in disease treatment.

## Graphical abstract

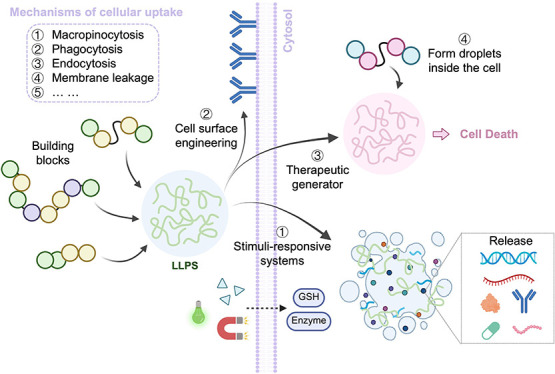

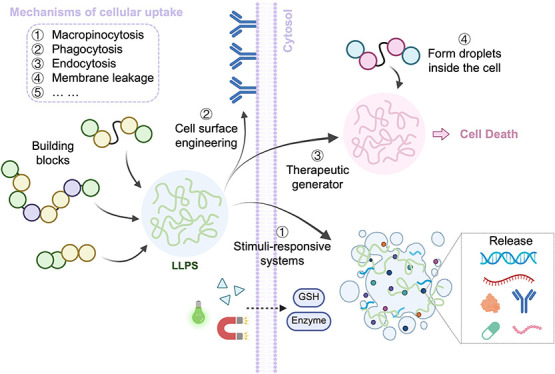

## 1 Introduction

Coacervates are dense liquid compartments that form through liquid–liquid phase separation (LLPS) in aqueous environments. Bungenberg de Jong and Kruyt first proposed the concept of coacervation, which originated from the Latin word *acervus*, meaning aggregation.^1^ LLPS is divided into segregative LLPS and associative LLPS, and the latter is also called complex coacervation. The droplets formed by complex coacervation and simple coacervation are referred to as coacervates.^2^ Complex coacervates typically consist of two different compounds, while simple coacervates are formed from a single component. When coacervation occurs, molecules spontaneously separate into a dense phase and a dilute phase. The dense phase is enriched with constituent molecules, forming coacervate droplets that segregate from the aqueous solution, while the surrounding dilute phase contains only a low concentration of molecules. Various noncovalent interactions, such as hydrogen bonding, π-π stacking, cation-π interactions, electrostatic interactions, and hydrophobic interactions, among others, drive this process.^3^ Unlike oil-in-water emulsions, where oil and water represent distinct phases, in the LLPS system, water serves as the continuous phase both inside and outside coacervates.^2^ Furthermore, coacervates are membraneless, enabling them to exchange solutes from the internal and external environments dynamically, and therefore, they are prone to ripening and fusion. This characteristic makes them suitable for constructing models of protocells.^4^

To further understand the mechanisms of coacervation and mimic intracellular compartments, researchers have explored a range of functional molecules, including proteins,^5^ nucleic acids,^6^ synthetic polymers,^7^ short peptides and their derivatives,^8–10^ surfactant-based amphiphilic compounds,^11–14^ and other small molecules.^15–19^ Among these, peptides and peptide derivatives have emerged as promising building blocks for biomedical materials due to their excellent biocompatibility and diversity. The peptide backbone, composed of diverse amino acid residues, provides a relatively simple and stable chemical structure. Functional groups can be introduced into the side chain of peptides to modulate phase separation behavior, guest molecule recruitment, and the physicochemical and catalytic properties of encapsulated cargo.^20^ One of the goals for peptide-based coacervate is to identify minimal motifs that can mimic compartments. An example was N-carboxybenzyl-protected phenylalanine dipeptide motifs (Z-FF) that could turn into thermodynamically favorable nanofibrils from liquid droplets over time.^21^ Caire da Silva et al.^22^ designed a diphenylalanine capped with a methoxy group (FF-OMe) that exhibited LLPS behavior and acted as a synthetic membraneless organelle and microreactor with biomimetic features and bioorthogonal catalytic capabilities. A combination of molecular dynamics simulations and experimental validation showed that the simple dipeptide Gln-Trp (QW) can form liquid droplets.^23^ In addition to dipeptides, simple aromatic groups containing amino acids, including 9-fluorenylmethoxycarbonyl (Fmoc)-Ala, Fmoc-His, Fmoc-Pro, Fmoc-Leu,^21^ were also capable of evolving from liquid droplets to thermodynamically favorable nanofibrils. Sato et al. reported the crystallization process of Fmoc-Lys after LLPS in DMSO-water mixtures.^24^ Elastin-like peptide (ELP) is another self-coacervate peptide derived from the hydrophobic domain of tropoelastin that has garnered considerable attention,^25^ although it is not classified as the LMWC. ELP consists of repetitive pentapeptide motifs with the sequence Val-Pro-Gly-Xaa-Gly (VPGXG), where Xaa represents any amino acid except proline. Owing to its thermoresponsive behavior, ELP has emerged as a promising candidate for various biomedical applications, including drug delivery,^26^ wound healing,^27^ and metal scavenging agents.^28^

The sticker-spacer model represents one of the simplest models for describing the formation of protein-mediated biomolecular condensates.^29,30^ LLPS is driven by attractive interactions between different amino acid residues within peptides, such as those between oppositely charged or aromatic residues, often referred to as stickers. To ensure that coacervates retain sufficient hydration and remain liquid-like, the remaining residues are typically polar and lack secondary structure, termed spacers. Phase-separating molecules normally achieve a balance between attractive interactions and hydration. These derivatives are usually designed to form coacervates under physiological conditions. A pioneering example developed by Spruijt et al.^31^ demonstrated that FFssFF (ss, disulfide) dipeptide derivatives showed a well-balanced combination of stickers and spacers, resulting in the formation of typical liquid droplets mainly driven by π-π stacking. Based on this work, they later reported a tyrosine-rich peptide (YFsFY),^32^ which transformed from droplets into stable membrane-enclosed protocells through enzymatic oxidation and cross-linking at the coacervate interface. Besides simple coacervates, complex coacervates can also be designed based on this model. Sticker-spacer type peptides DGR_5_Cs-sCR_5_GD formed coacervates with nicotinamide adenine dinucleotide phosphate (NADPH) by electrostatic interactions.^33^

In recent years, the design strategy and principle of phase-separating molecules have been extended to small molecules, and many sticker-spacer-type small-molecule-based phase-separating systems have been reported. For instance, pyrene-based photoresponsive phase-separating molecules enabled transmembrane delivery of biomacromolecules,^16^ and Staudinger reaction-responsive LLPS systems achieved targeted degradation of both membrane and intracellular proteins.^17^ Additionally, spiropyran-derived coacervates significantly enhance the efficiency of reactive oxygen species (ROS) generation, thereby potentiating antitumor efficacy.^18^ Besides sticker-spacer type compounds, amphiphilic molecules show high potential to form coacervates under specific aqueous conditions, especially at higher concentrations.^34–37^ For example, the cationic single-chain surfactant, *N*-methylephedrinium bromide (DMEB), underwent spontaneous LLPS in the presence of salts, such as NaCl.^38^ George et al. reported a naphthalene diimide (NDI)-based molecule containing boronic acid and ammonium groups, which formed metastable droplets in its zwitterionic state under alkaline conditions.^39^ Here, we collectively call short peptides and small molecules as low-molecular-weight compounds (LMWCs), and this perspective will focus on coacervates formed by LMWCs.

Compared to proteins and polymers, LMWC-based coacervates provide a simplified and modular alternative for modeling biomolecular condensates. Their key advantages include a modular molecular design, ease of synthesis, and the ability to incorporate diverse stimuli-responsive functionalities. LMWC-based coacervate droplets, ranging in size from nanometers to micrometers, represent highly promising vehicles for cargo delivery. First, coacervate droplets serve as a versatile platform for encapsulation and intracellular delivery of various therapeutic agents. Cargos are encapsulated within droplets through weak noncovalent interactions, enabling the incorporation of a wide range of therapeutics while avoiding the hassle of covalent conjugation, which might alter their bioactivity. The rapid recruitment of cargo in aqueous environments is particularly beneficial for preserving the bioactivity of macromolecules, including therapeutic drugs, hydrophilic proteins, and enzymes. Partial droplets exhibiting lower fluidity could protect the carrier from degradation, making them suitable for transporting easily degradable biomolecules, such as RNAs. Second, the relatively large size of these droplets facilitates a high cargo loading capacity. Coacervates provide a compartmentalized environment that concentrates reactants, thereby improving reaction rates. Furthermore, the ability of coacervate droplets to undergo reversible self-assembly endows them with high dynamicity and excellent tunability, allowing for the design of stimuli-responsive systems that can adapt to environmental triggers such as enzymes, redox conditions, light, or disease states. This characteristic makes coacervate droplets suitable for controlled drug delivery. Interestingly, the cellular uptake mechanisms of coacervates are complex and diverse, distinguishing them from conventional delivery vehicles, such as polymer, liposomes, polymersomes, and silica nanoparticles. As research progresses, scientists are gradually uncovering the mysteries of coacervates. This perspective highlights the transmembrane mechanisms of these LMWC-based coacervates and their potential in the delivery of macromolecular therapeutics and the treatment of diseases. Additionally, we discuss the opportunities and challenges associated with these delivery systems.

## 2 Mechanisms of Cellular Uptake of LMWC-Based Coacervates

Coacervates can serve as liquid capsules for cargo delivery due to high loading capacity, low cytotoxicity, and high cellular uptake efficiency. Such coacervate droplets with membrane-penetrating properties have great potential as intracellular delivery vehicles. However, coacervate droplets have limited internalization pathways into the cell. There are two most common pathways for larger objects (>1 μm) to enter cells: macropinocytosis^40^ and phagocytosis.^41^ Cholesterol also plays an important role in the interaction between coacervates and membranes, affecting membrane deformation, and many coacervate transmembrane pathways are cholesterol dependent. (Table 1)

**Table 1:** Cellular Uptake Mechanisms for LMWC-Based Coacervates

uptake mechanism	merits	potential drawbacks
**macropinocytosis** **& pha** **gocytosis** (for larger droplets)	- **high efficiency for large carriers**: ideal for internalizing micrometer-sized coacervates	- **limited control**: a relatively nonspecific process
	- **bypasses lysosomal degradation**: direct cytosolic delivery	- **membrane disruption**: potential cytotoxicity
**cholesterol-dependent pathways/lipid rafts**	- **enhanced binding** **& uptake**: cholesterol increases membrane rigidity	- **dependence on membrane composition**: variability in cholesterol content
	- **specific interactions**: aromatic and histidine residues in peptides can specifically interact with cholesterol	
**direct membrane leakage/perforation**	- **complete lysosome bypass**: ideal for cargoes highly susceptible to lysosomal degradation (e.g., siRNA, certain proteins)	- **high cytotoxicity risk**: sustained membrane damage can lead to cell death
	- **rapid entry**: does not rely on the slower endocytic machinery	- **lack of control**: the process is inherently disruptive and difficult to regulate spatially and temporally
**endocytosis** (e.g., clathrin-mediated; for smaller droplets/nanodroplets)	- **lower cytotoxicity**: a more natural, less disruptive cellular process	- **lysosomal degradation risk**: Therapeutic cargo can be destroyed.
	- **potential for targeted delivery**: e.g., targeted protein degradation	- **requires efficient endosomal escape**: needs an additional mechanism to release cargo into the cytosol
**hybrid mechanism** (e.g., phagocytosis + macropinocytosis)	- **robust uptake**: potentially making uptake more efficient and reliable across different cell types and droplet states	- **difficult to predict and control**: the involvement of multiple pathways makes it challenging to engineer coacervates to favor one specific, optimal route
	- **comprehensive model**: more accurately reflects the observed complexity of coacervate internalization, involving filopodia engagement and membrane remodeling	

Recently, increasing evidence has demonstrated that macropinocytosis and phagocytosis are likely the major pathways for the uptake of coacervate droplets. This concept was pioneered by Futaki et al.,^42^ who successfully combined the macropinocytosis inducer (peptide derived from stromal-derived factor 1α, SN 21) with membrane-lytic peptides to deliver diverse macromolecular cargoes, including functional siRNA, antibodies, Cre recombinase, and artificial transcription regulators. In subsequent work,^43^ they achieved cytosolic delivery of negatively charged (Alexa-label) immunoglobulin G (IgG) through complexation with cationic lytic peptides FcB­(L17E)_3_ to form coacervate microdroplets (CMs). CMs were able to access the cell membrane and the involvement of macropinocytosis-like uptake mechanisms. The attachment of liquid droplets to the cell membrane induced actin reorganization and membrane ruffling, possibly leading to transient membrane permeabilization. Finally, successful cytosolic delivery of Alexa-IgG leads to interaction with the cellular targets (Figure 1a). Membrane leakage is another pathway for coacervate to cross the cell membrane. This finding was reported by Wang et al., who developed a novel phosphopeptide construct (KYp) composed of an anticancer peptide [KLAKLAK]_2_ (K) and a phosphatase-responsive phosphorylated tyrosine residue (Yp).^44^ Upon encountering alkaline phosphatase (ALP), KYp underwent enzymatic dephosphorylation, triggering its in situ self-assembly into nanoparticles. This transformation induced ALP aggregation and initiated protein–lipid phase separation at the plasma membrane, ultimately compromising membrane integrity and enhancing cellular permeability. Notably, the KYp-ALP aggregates circumvented conventional endocytic pathways, instead exploiting membrane leakage for cellular entry while avoiding lysosomal degradation. Smaller nanoparticles could still enter cells via caveolae-mediated endocytosis. This dual-mode internalization mechanism suggests that the pathway by which coacervates enter cells may be related to their size: large coacervates prefer macropinocytosis, while smaller coacervates prefer endocytosis (Figure 1b).

**Figure 1:**
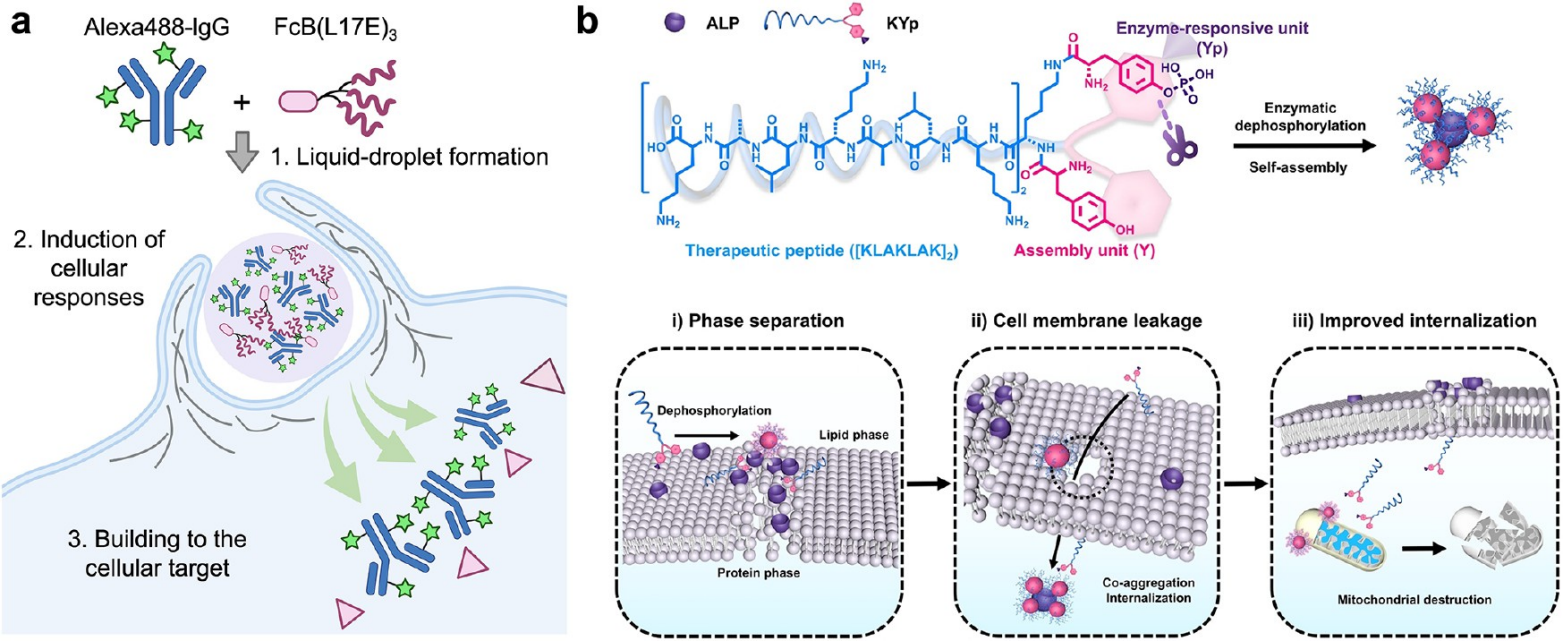
Cellular internalization mechanisms of liquid droplets. a. Liquid droplet formation and facile cytosolic translocation of Alexa488-IgG in the presence of attenuated cationic amphiphilic lytic peptides FcB­(L17E)_3_. b. Phosphatase (ALP) responsive peptide (KYp) and its self-assembly on the cell membrane. The internalization of ALP and KYp aggregates leads to leakage of the cell membrane, finally enhancing internalization. Reproduced with permission from ref 44. Copyright 2021 Wiley-VCH GmbH.

In 2022, Spruijt et al. employed a liposome model system to investigate the interactions between coacervates and phospholipid bilayers.^45^ Their study revealed that electrostatic interactions played a pivotal role in governing three key processes: (1) coacervate wetting on membrane surfaces, (2) subsequent membrane deformation, and (3) eventual endocytosis of coacervates into liposomes. They designed liposomes with tunable surface charges by varying the composition of POPC (1-palmitoyl-2-oleoyl-*sn*-glycero-3-phosphocholine), cholesterol, and charged lipids (either cationic DOTAP or anionic POPG), and systematically studied these interactions using complex coacervates with tunable surface charge as a model, such as spermine/polyU. An attractive droplet-membrane interaction drives successful endocytosis. In addition, the larger coacervates were found to partially wet the liposomes, while the smaller coacervates were engulfed (Figure 2a). This work established a crucial physical framework for understanding the interactions between biomolecular condensates and membranes. Additionally, it advanced potential applications in synthetic biology and the development of innovative intracellular delivery strategies. However, while liposomes provide a valuable reductionist model, they lack the complexity of living cells, underscoring the need for future in-cell validation of these findings. Cholesterol also played an important role in mediating coacervate internalization. Song et al. developed a fluorescent biosensor TPE-(RRASL)_n_ by conjugation of an aggregation-induced emission luminogen (AIEgen) tetraphenylethene (TPE) with phase-separating peptides (RRASL)_n_ for intracellular RNA visualization.^46^ The probe formed coacervate droplets when it interacted with RNA. Coacervates efficiently penetrated cells through a cholesterol-dependent pathway and involved lipid rafts, as evidenced by their sensitivity to methyl-β-cyclodextrin (MβCD); however, the exact mechanism had not been studied in detail.

**Figure 2:**
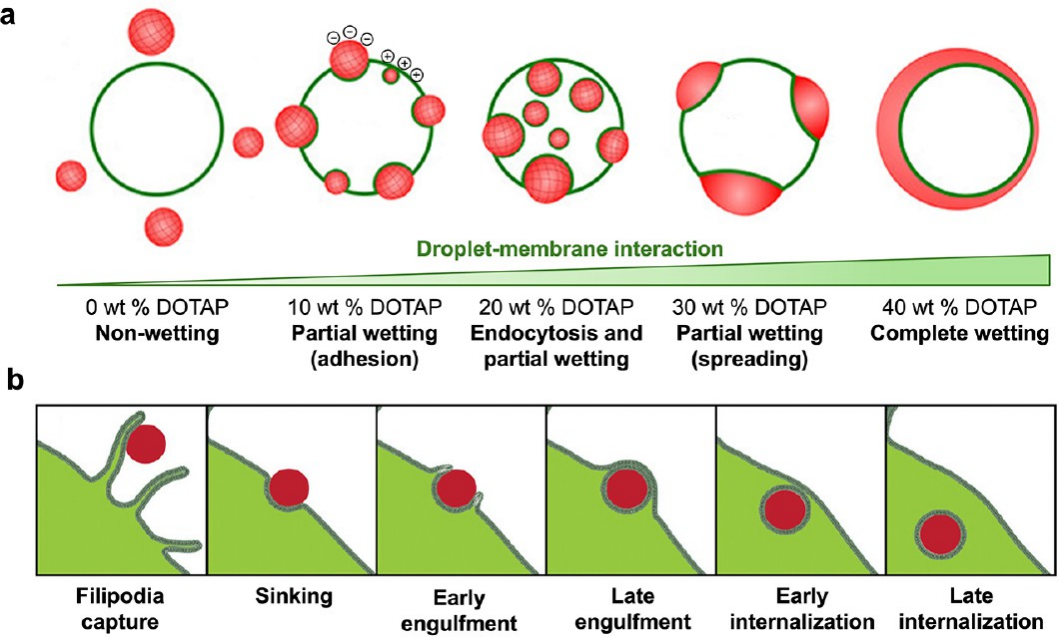
Interaction dynamics of coacervates with liposome or cell membrane. a. Interaction of POPC/DOTAP liposomes and spermine/polyU coacervates for different DOTAP fractions. Reproduced from ref 45. Copyright 2022 American Chemical Society.b. Schematics of different stages of HBpep-SP coacervate uptake. Reproduced from ref 53. Available under a CC-BY license 4.0. Copyright 2024 Wiley-VCH GmbH.

In addition to complex coacervates, significant progress has been made in understanding the cellular internalization mechanisms of simple coacervates, which are often enriched in aromatic amino acids such as phenylalanine (Phe), tyrosine (Tyr), and tryptophan (Trp). These coacervates are frequently localized at lipid membrane interfaces^47^ and are important in protein–lipid bilayer interactions,^48^ including cholesterol binding^49^ and peptide/protein membrane anchoring.^50^ Notably, modulating the properties of aromatic rings has been shown to enhance the cellular uptake of simple coacervates.^51^ A particularly insightful study by Cardenas et al.^52^ examined the interaction of the HB*pep* peptide with lipid bilayers at physiological pH. They found that HB*pep* preferentially bound to membrane surfaces without disrupting bilayer integrity, highlighting the importance of aromatic residues (tyrosine and tryptophan) and histidine in mediating interactions with the β-hydroxyl group of cholesterol. These observations suggested that cholesterol-peptide interactions were significantly important for coacervate-membrane binding and subsequent cellular internalization, but this needed further confirmation. Recently, Miserez et al.^53^ systematically captured and characterized key stages in the cellular internalization of histidine-rich coacervates (HB*pep* and HB*pep*-SP). The internalization process was initiated when filopodia-like membrane protrusions engaged the coacervate droplets through a phagocytosis-like recognition mechanism. Cholesterol played a critical role in coacervate binding and recognition by modulating membrane rigidity. Increased cholesterol content could enhance bending rigidity, facilitating membrane binding, which could improve coacervate-membrane interactions and internalization efficiency. High-resolution imaging techniques, including HAADF-STEM and SEM, provided structural insights into the internalization dynamics. Coacervate internalization involved progressive droplet deformation followed by topological rupture of the plasma membrane, leading to their sink, engulfment, and intracellular entry. This process was driven by energy-dissipative cytoskeletal remodeling. Remarkably, the internalization pathway exhibited a unique hybrid mechanism, incorporating characteristic features of both phagocytosis and macropinocytosis. This dual-pathway involvement provided a comprehensive framework for understanding coacervate-mediated intracellular delivery (Figure 2b). Besides, Miserez’s group also found that the uptake efficiency was related to the peptide sequence. Stronger membrane adhesion via electrostatic interactions with positive charge residues led to faster uptake.^54^

Although significant progress has been made in recent years regarding the internalization pathways of LMWC-based coacervates, our current understanding remains merely the tip of the iceberg. The transmembrane mechanisms of coacervates are complex and diverse, owing to the multiple driving forces governing their coacervation, which may lead to varied interactions between coacervates and lipid membranes. Furthermore, coacervate droplets exhibit a broad size distribution, and droplets of different size ranges may employ distinct membrane-crossing pathways. Currently, there is a lack of rapid and straightforward methods to track the transmembrane pathways of coacervate droplets, with research primarily relying on microscopy and inhibitor assays. The development of specialized probes for tracing coacervate transmembrane mechanisms would significantly advance the study of cellular uptake processes involving coacervates. For example, the recruitment of microenvironment-sensing probes into coacervates might provide a means to differentiate between uptake pathways. A mechanism like direct membrane leakage results in minimal alteration of the coacervate interior. In contrast, processes involving interactions with the cell membrane, such as endocytosis, might induce a more crowded microenvironment, allowing the probe to detect this alteration. In addition, the cytotoxicity of coacervates may be associated with their uptake mechanisms. In the internalization process, coacervates can cause membrane deformation and perforation, and sustained, widespread membrane damage may result in cell damage.

## 3 Applications in Therapy

Coacervate droplets have been extensively studied and applied in disease treatment, particularly in the context of stimuli-responsive droplet formation or disassembly to release therapeutic agents. These droplets have also been utilized for cellular surface engineering. Furthermore, due to their ability to enhance local concentrations, coacervates can accelerate reaction rates, which has been leveraged to boost the production of anticancer species and improve therapeutic efficacy. A detailed discussion of selected examples will be given below (Table 2).

**Table 2:** LMWC-Based Coacervates for Intracellular Delivery

LMWC system	cellular uptake mechanism	driving force for LLPS	intracellular cargo release mechanism
cationic lytic peptide (FcB(L17E)_3_) + Alexa-IgG^43^	macropinocytosis; membrane ruffling and transient permeabilization	electrostatic interactions, π–π stacking, and cation−π interactions	
phosphopeptide (KYp)^44^	direct membrane leakage (for large aggregates); caveolae-mediated endocytosis (for smaller nanoparticles)	enzymatic (ALP) dephosphorylation triggers self-assembly via enhanced hydrophobicity and intermolecular interactions.	
spermine/PolyU Coacervates^45^	endocytosis (in model liposomes); dependent on electrostatic droplet–membrane attraction	electrostatic interactions	model system; release not studied in cells
TPE-(RRASL)_n_ ^46^	cholesterol-dependent pathway; involves lipid rafts	π–π stacking, cation−π interactions	not for release. Combine with intracellular RNA for live-cell imaging
histidine-rich peptides (HB*pep*, HB*pep*-SP, and variants)^53,54,68–71^	phagocytosis and micropinocytosis; cholesterol-dependent lipid rafting	π–π stacking, cation−π interactions, hydrophobic interactions, hydrogen bonding	redox (GSH)-triggered disassembly
sulfatase-responsive peptide (Y^SO4^F)^55^	lysosome-mediated uptake pathway for peptide Y^SO4^F	π–π stacking	they function by coalescing with and disrupting stress granules, not by releasing a drug cargo
thrombin-cleavable peptide + heparin^56^		electrostatic interactions	enzyme (thrombin)-triggered disassembly
mussel peptides (GG23*/GK16)^58^	designed for oral delivery	Dopa-mediated multivalent interactions, including bidentate hydrogen bonds, π–π stacking, and cation−π interactions	enzyme (trypsin)-triggered disassembly
PPFM^16^	energy-dependent endocytosis; direct entry into the cytosol	π–π stacking, hydrophobic interactions	photoresponsive disassembly
PEGylated HBpep-K^SP^ ^72^	clathrin-mediated endocytosis (for nanodroplets, CNs); macropinocytosis (for microdroplets, CMs)	π–π stacking, cation−π interactions, hydrophobic interactions; PEG chain length controls droplet size	redox (GSH)-triggered disassembly
erythrocyte-membrane coated coacervates (EMCoac@miR)^73^	cholesterol-dependent lipid raft	electrostatic interactions	1) lipid raft-mediated membrane fusion; 2) GSH-triggered cytosolic disassembly
enantiomeric peptides (GHGXY)_4_ ^74^	energy-dependent macropinocytosis	cation−π interactions, π–π stacking, hydrophobic interactions; Chirality does not affect LLPS.	
dimeric CWWWRRGD^75^		CATion−π interactions, π–π stacking, hydrophobic interactions, electrostatic interactions, hydrogen bonding	GSH-responsive
dextran nanogel-shielded coacervates (DNScs)^76^	endocytosis	electrostatic interactions	GSH-responsive
NADPH/DGR_5_CssCR_5_RGD coacervates^33^		cation−π interactions, π–π stacking, electrostatic interactions	glucose-6-phosphate dehydrogenase (G6PD)-driven NADPH oxidation and GSH-responsive
DgHBP-2 ^77^		hydrophobic interactions, π–π stacking	glucose-responsive
peptide amphiphile (PA–PBA) + dasiglucagon^79^		electrostatic interactions	glucose-concentration-dependent dissolution
Staudinger reaction-responsive coacervates (SR-Coa)^17^		hydrophobic interactions, π–π stacking, hydrogen bonding	Staudinger reaction-triggered dissolution
magnetic coacervates (DMCs)^81^	energy-independent mechanism, not based on endocytosis	hydrogen bonding, π–π interactions	magnetically and thermally triggered release
FA-PC@MPNs^82^	receptor-mediated targeted uptake	hydrogen bonding, hydrophobic and electrostatic interactions	GSH-responsive and lysosomes
Fmoc-Lys-Gly-Dopa–OH (KGdelta)^84^	not for uptake; coacervates coat the cell surface via Fe^3+^ coordination	π–π stacking, electrostatic interactions, and hydrogen bonding	not for release; the system is used to display antibodies (e.g., trastuzumab) on the surface of NK cells for targeted killing
spiropyran coacervates (SP-C)^18^	accumulate in tumors; cellular uptake mechanism not detailed	hydrophobic interactions	not a delivery vehicle; the coacervates themselves are the therapeutic agent, generating ROS upon light illumination
CSC-GHT^85^	clathrin-mediated endocytosis (CME)	electrostatic interactions	not a delivery vehicle; concentration of biomolecules to generate chemical gradients

### 3.1 Stimuli-Responsive Systems

#### 3.1.1 Enzyme

Enzymatic regulation of biomolecular coacervates becomes a key research area because enzymatic pathways are essential for controlling the assembly, disassembly, and functional behavior of these condensates. Certain enzymes overexpressed in cancer cells may further enable the *in situ* formation of coacervates within tumor cells, thereby achieving targeted coacervate modulation. For example, Yu et al. designed a sulfatase-responsive sulfated tyrosine-rich peptide, Y^SO4^F. Mixture of Y^SO4^F and the stress granules (SGs)-targeting ligand FGDF conjugated Y^SO4^F (FGDF-Y^SO4^F) gave m-Y^SO4^F-L^SG^, which undergoes sulfatase-triggered LLPS to generate droplets (d-YF-L^SG^).^55^ Under sorafenib treatment, these droplets coalesced with SGs via specific interactions between FGDF and the SG core protein G3BP2. This disrupted SG-mediated cytoprotective functions, ultimately promoting caspase-3-dependent apoptosis (Figure 3).

**Figure 3:**
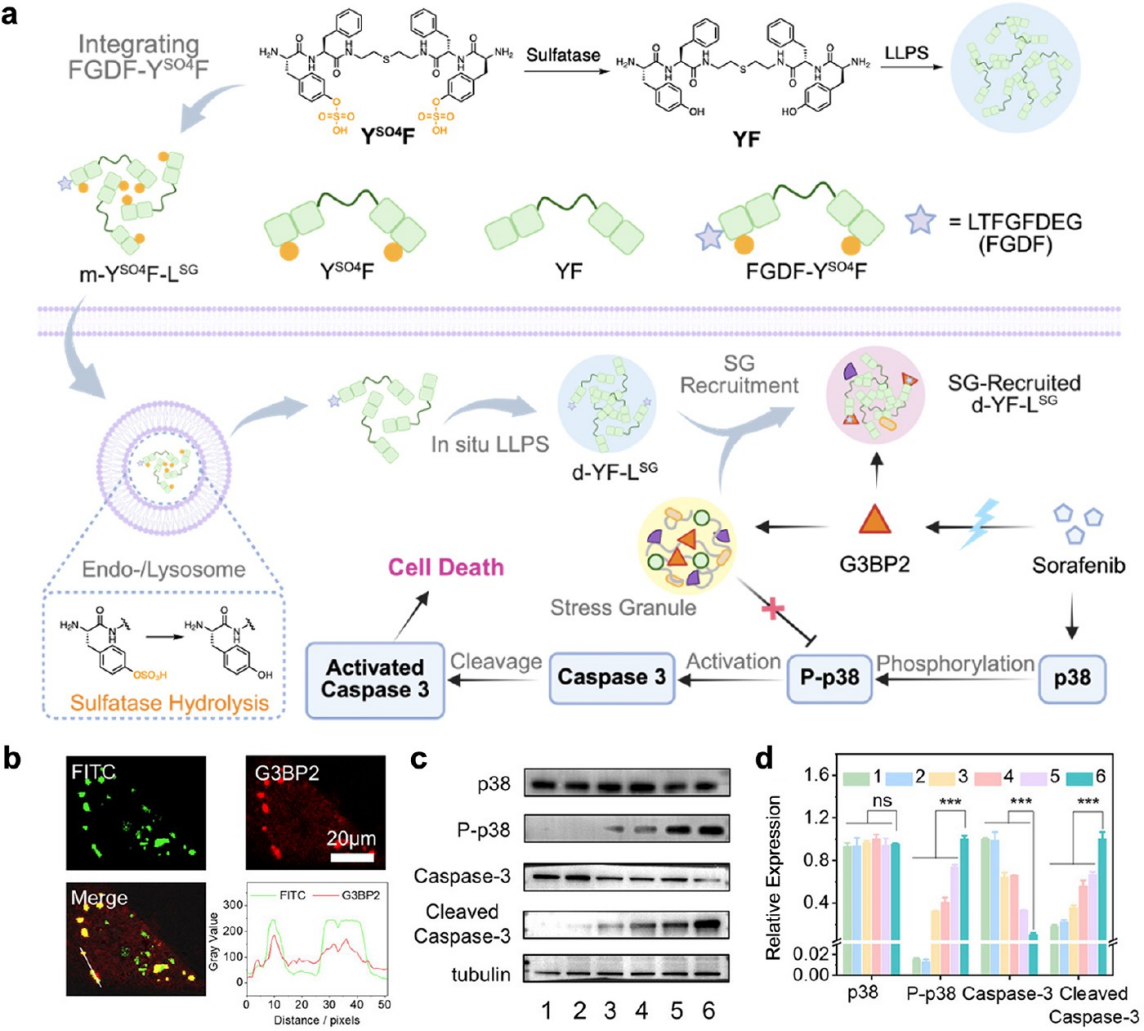
*In situ* formation of coacervate droplets and their role in targeting stress granules for cancer therapy. a. Schematic illustration of in situ formation of coacervate droplets in living cells and targeting stress granules (SGs) for cancer chemotherapy, combining with sorafenib. b. Confocal images of immunofluorescence G3BP2-stained A549 cells treated withm-Y^SO4^F-L^SG^ for 12 h. c, d. Western blot assays (c) and quantitative data (d) of the intracellular levels of p38, P-p38, caspase-3 and cleaved caspase-3 in A549 cells treated with PBS (1),m-Y^SO4^F-L^SG^ (2) for 24 h, or sorafenib (3) for 12 h, and treated byd-YF-L^SG^ (4),FGDF-Y^SO4^F (5), orm-Y^SO4^F-L^SG^ (6) for 12 h and coincubated with sorafenib for additional 12 h. Reproduced with permission from ref 55. Copyright 2025 Wiley-VCH GmbH.

A thrombin-triggered coacervate was reported by Jokerst and co-workers.^56^ In detail, nanocoacervates (NC) were engineered by incorporating a thrombin-cleavable LVPR sequence between two YR units in the peptide, which then self-assembled with heparin. Tannic acids (TAs) formed supramolecular networks within coacervates, improving stability and maintaining spherical morphology under vacuum. Fluorescence recovery and anticoagulation assays confirmed that NC-TAs exhibited a thrombin-dependent disassembly, releasing heparin. In whole blood, NC-TAs inhibited clot formation compared with free heparin (Figures 4 **a-d**).

**Figure 4:**
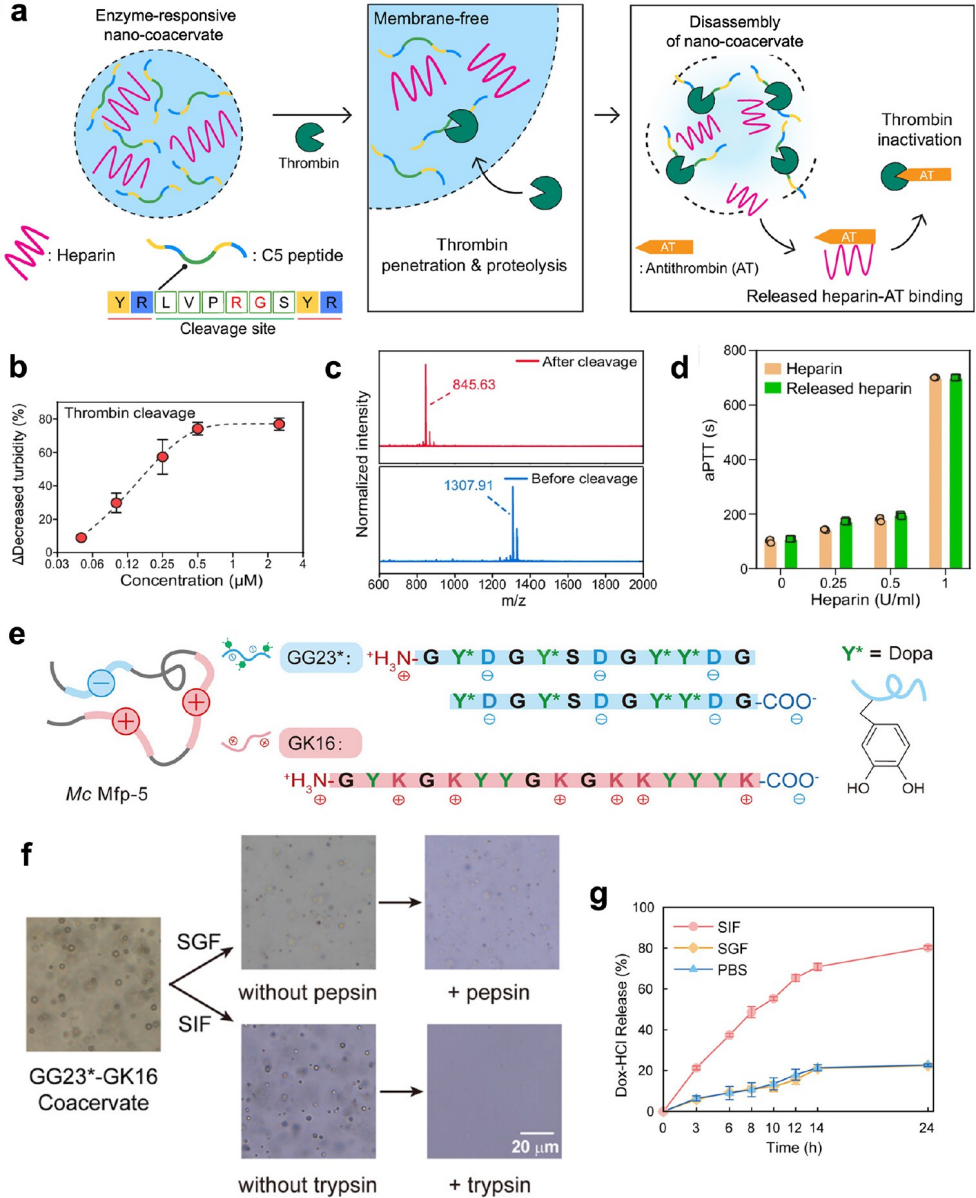
Enzyme-triggered disassembly of coacervates for drug release. a. Schematic illustration of thrombin-mediated proteolytic disassembly of nanocoacervates for heparin release. The released heparin binds antithrombin, inducing thrombin inactivation. b. Decreased turbidity of nanocoacervates with different thrombin concentrations. c. MALDI-MOF data of peptides before and after thrombin cleavage. d. The activated partial thromboplastin time (aPTT) test of heparin and released heparin from the disassembly of nanocoacervates. Reproduced from ref 56. Available under a CC-BY license 4.0. Copyright 2024 Nature Publishing Group. e. Sequence of GG23* and GK16 peptide. (* = Tyr modified into Dopa) f. Microscopy images of GG23*-GK16 coacervate remain stable in simulated gastric fluid (SGF) while exhibiting enzyme-triggered disassembly upon the addition of trypsin in simulated intestinal fluid (SIF). g. Drug release kinetics of Dox-HCl encapsulated in GG23*-GK16 coacervates. Reproduced from ref 58. Copyright 2025 American Chemical Society.

The coacervate-mediated delivery platform also demonstrates excellent utility in potentiating the pesticidal activity of agrochemical formulations.^57^ Agrochemicals could be delivered by nucleic acid-peptide coacervates (NPCs) formed by negatively charged ssDNA and positively charged poly-l-lysine (PLL), which were able to encapsulate agrochemicals emamectin benzoate (EB) and abscisic acid (ABA) with 80% and 33.5% efficiency, respectively. NPCs protected photosensitive EB and ABA from UV degradation, and higher ssDNA content enhanced protection. The coacervates underwent disassembly by endogenous nucleases and other active substances within the pest’s body, triggering the rapid release of EBs and enhancing pesticidal efficacy.

Mussel foot protein 5 (Mfp-5) undergoes LLPS in acidic environments. Inspired by this phenomenon, Yu et al.^58^ developed a complex coacervate system composed of two engineered peptides: GG23* (GYDGYSDGYYDGYDGYSDGYYDG) and GK16 (GYKGKYYGKGYKKYYYK), where * denotes tyrosine modified to Dopa. The Dopa modification significantly reduced the critical concentration required for coacervation while enhancing the salt resistance of the resulting coacervates. Oral drug delivery is often the preferred administration route due to its convenience and high patient compliance.^59^ However, many therapeutic molecules are prone to degradation and inactivation upon exposure to the highly acidic gastric environment.^60^ Encapsulating these drugs within GG23*-GK16 coacervates could improve their stability during gastric transit, thereby enhancing therapeutic efficacy. Results showed GG23*-GK16 coacervates remained stable in simulated gastric fluid (SGF), retaining encapsulated cargo, while enabling trypsin-triggered release in simulated intestinal fluid (SIF). This controlled release mechanism arose from the complex interaction network within the coacervate, which hindered the formation of pepsin-substrate complexes and reduced cleavage efficiency.^61^ In contrast, the specificity of trypsin was attributed to the electrostatic attraction between the negatively charged aspartate (Asp) in its binding pocket and positively charged amino acids,^62^ leading to the disintegration of coacervate. The GG23*-GK16 coacervate platform was particularly valuable for drugs susceptible to gastric degradation or metabolism, as it ensured the delivery of a higher fraction of active ingredients to the small intestine for absorption, thereby improving bioavailability and therapeutic outcomes (Figures 4 **e-g**).

Furthermore, matrix metalloproteinase 9 (MMP-9) promoted a solid-to-coacervate transition of TPE-(RRASL)_2_-GPLGLAGFY/hyaluronic acid (HA) complex coacervate, resulting in improved infiltration of the coacervates into Hey cells and tumor spheroids.^63^ KYp underwent ALP-triggered dephosphorylation, leading to self-assembly into nanoparticles, which subsequently induced protein–lipid phase separation at the plasma membrane, as described in the previous section.^44^

#### 3.1.2 Light

Light-mediated modulation provides a convenient and precise approach for controlling the spatiotemporal behavior of coacervate droplets, enabling the dynamic regulation of phase separation and material properties. Many photocontrolled phase-separating molecules have been reported. For example, an aggregation-induced emission luminogen (AIEgens), tetraphenylethylene (TPE), conjugated to RNA-binding repeats of (RRASL)_*n*_ (*n* = 1, 2, 3), offered a versatile tool for studying RNA-targeted diagnostics. Above the minimum effective concentration (MEC), TPE-(RRASL)_*n*_ demonstrated efficient cellular penetration into human gallbladder carcinoma cells (SGC-996). It translocated into the cell nucleus and illuminated intracellular RNA, indicating that TPE-(RRASL)_*n*_ has the potential to serve as an RNA sensor for live-cell imaging (Figures 5 **a-b**).^46^ Coacervates formed by DNA and photoswitchable azobenzene cations (azoTAB) exhibited reversible disassembly/assembly under UV (365 nm) and blue light (450 nm) via trans–cis photoisomerization of azoTAB (Figure 5c).^64^ However, few light-controlled coacervate systems have been applied to the delivery of biomacromolecules. Our lab reported a photoresponsive, phase-separating fluorescent molecule (PPFM) that enabled noncovalent protein delivery with light-controlled cytosolic release.^16^ Norrish Type II reaction was introduced into molecules, and 405 nm light disintegrated droplets, releasing payloads into the cytosol. The PPFM system could recruit proteins of varying sizes and pI values, and proteins, including enzymes, could maintain activity after release. One example is saporin, a ribosome-inactivating protein that can be delivered into the cell and kill the cancer cell after release (Figures 5 **d-e**). However, the short wavelength of light irradiation and the release of thioaldehyde after photolysis might limit the application of the PPFM system *in vivo*. Nevertheless, longer-wavelength-responsive stickers normally exhibited stronger hydrophobicity, which would lead to aggregation and precipitation. Therefore, designing low-molecular-weight phase-separating delivery systems with milder light-responsive conditions remains a significant challenge.

**Figure 5:**
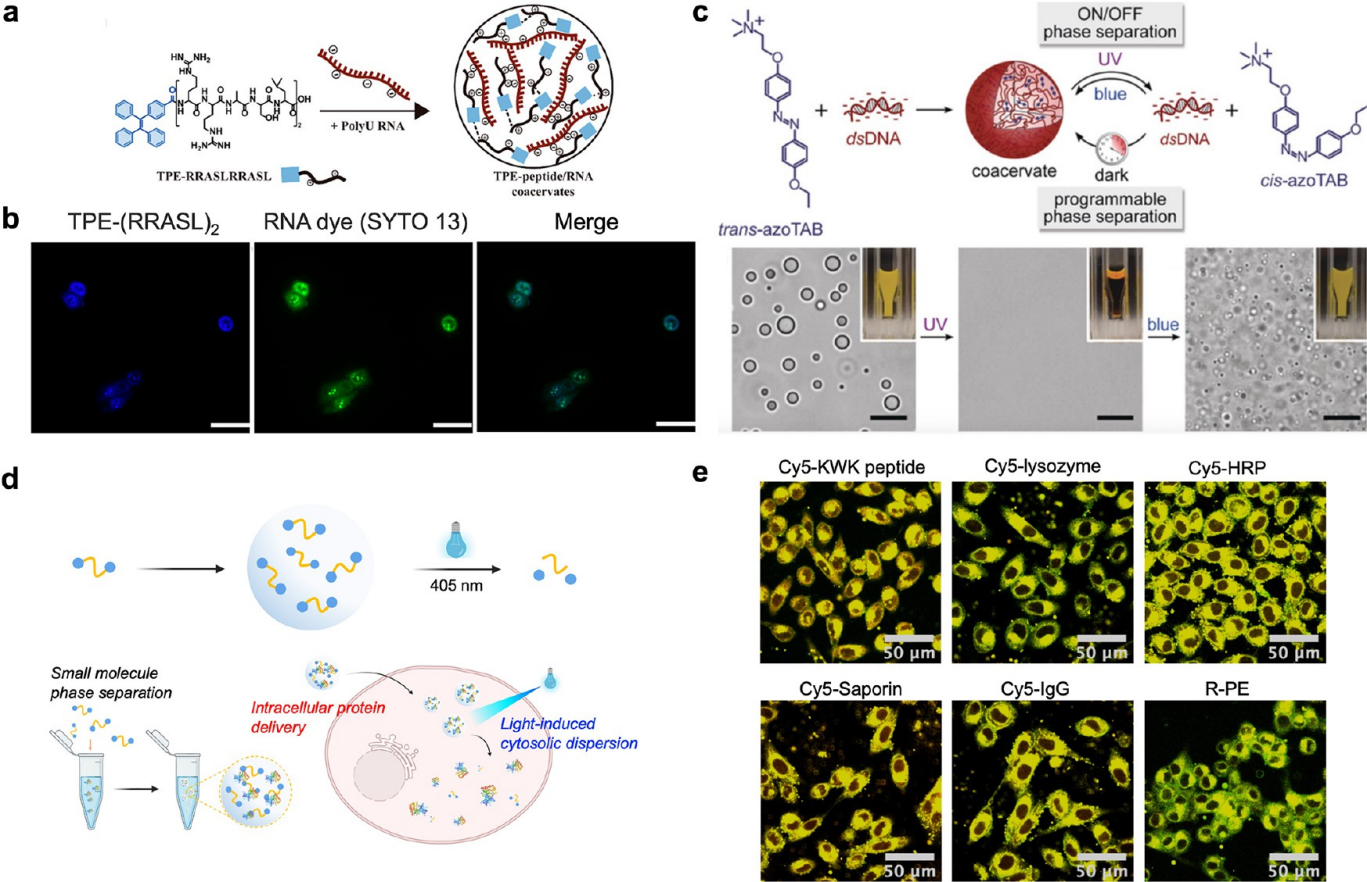
Light-Responsive Coacervates. a. Schematic illustration of TPE-(RRASL)_*n*_/polyU coacervates.b. Fluorescence microscopy images show that TPE-(RRASL)_2_ penetrates SGC-996 cells and translocates into the cell nucleus, where it colocalizes with the standard RNA dye, SYTO 13. Scale bars, 50 μm. Reproduced from ref 46. Copyright 2023 American Chemical Society. c. Schematic of the light-regulated coacervation between trans-azoTAB and dsDNA. The system undergoes reversible liquid–liquid phase separation, forming coacervates that disassemble under UV light (to cis-azoTAB) and reassemble under blue light. scale bars, 10 μm. Reproduced with permission from ref 64. Copyright 2019 Wiley-VCH GmbH. d. Schematic illustration of a photoresponsive, phase-separating fluorescent molecule (PPFM). e. Confocal microscopy images show that different peptides and proteins can be delivered to the cytosol of HeLa cells by PPFM coacervates after photo illumination. Reproduced with permission from ref 16. Copyright 2023 Wiley-VCH GmbH.

#### 3.1.3 Reduction–Oxidation Reaction (Redox)

The intracellular redox balance is essential for cellular function, including respiration, oxidative stress defense, signaling, ATP production, and protein activity.^65^ This regulation extends to biomolecular condensates, where redox states modulate their assembly and stability via oxidation-sensitive protein-nucleic acid interactions that govern functional properties.^66^ These principles have been harnessed to engineer redox-responsive synthetic coacervates,^67^ providing a platform to mimic cellular compartmentalization through controlled molecular interactions. By tuning redox conditions, these systems enable precise microenvironmental control for directing biochemical reactions and structural organization. Key redox-switchable moieties, such as disulfides, thioethers, and NADP+, incorporated into LLPS-driving molecules undergo reversible hydrophobicity changes upon redox stimulation, enabling programmable coacervate assembly and disassembly.

Typical examples of self-coacervation include histidine-rich squid beak proteins (HBPs), which were characterized by their low sequence complexity. Miserez’s group had made great efforts to study peptide sequence from HBP, composed of Gly-His-Gly-X-Tyr (GHGXY) repeats, where X was a variable residue, and their coacervation and membrane-crossing mechanisms in recent years. A representative example was HB*pep*-SR.^68^ The peptide formed coacervates, and the positive charge of the lysine side chain shifted the optimal LLPS pH from 7.5 to 9.0. This modulation enabled efficient recruitment and delivery of diverse biomolecular cargos into cells, including small peptides, enzymes, and mRNAs (mRNAs). Unlike conventional delivery systems, these payload-loaded coacervates bypassed classical endocytic pathways and entered the cytosol directly. Subsequently, the reducing cytosolic environment, mediated by glutathione (GSH), triggered the removal of lysine side-chain groups, resulting in the disassembly and release of the payload (Figure 6). Building on this platform, HB*pep*-SP coacervates have been further applied as a universal delivery vehicle for three types of CRISPR/Cas9 gene-editing tools, including pDNA, mRNA/sgRNA, and the ribonucleoprotein complex.^69^ These modalities are spontaneously recruited into peptide coacervates via simple mixing, outperforming even highly optimized commercial transfection reagents in both transfection and genome editing efficiency. It also enabled efficient and nontoxic intracellular delivery of omoMYC (a MYC inhibitor).^70^ Once inside, the GSH-triggered coacervate disassembly released omoMYC.

**Figure 6:**
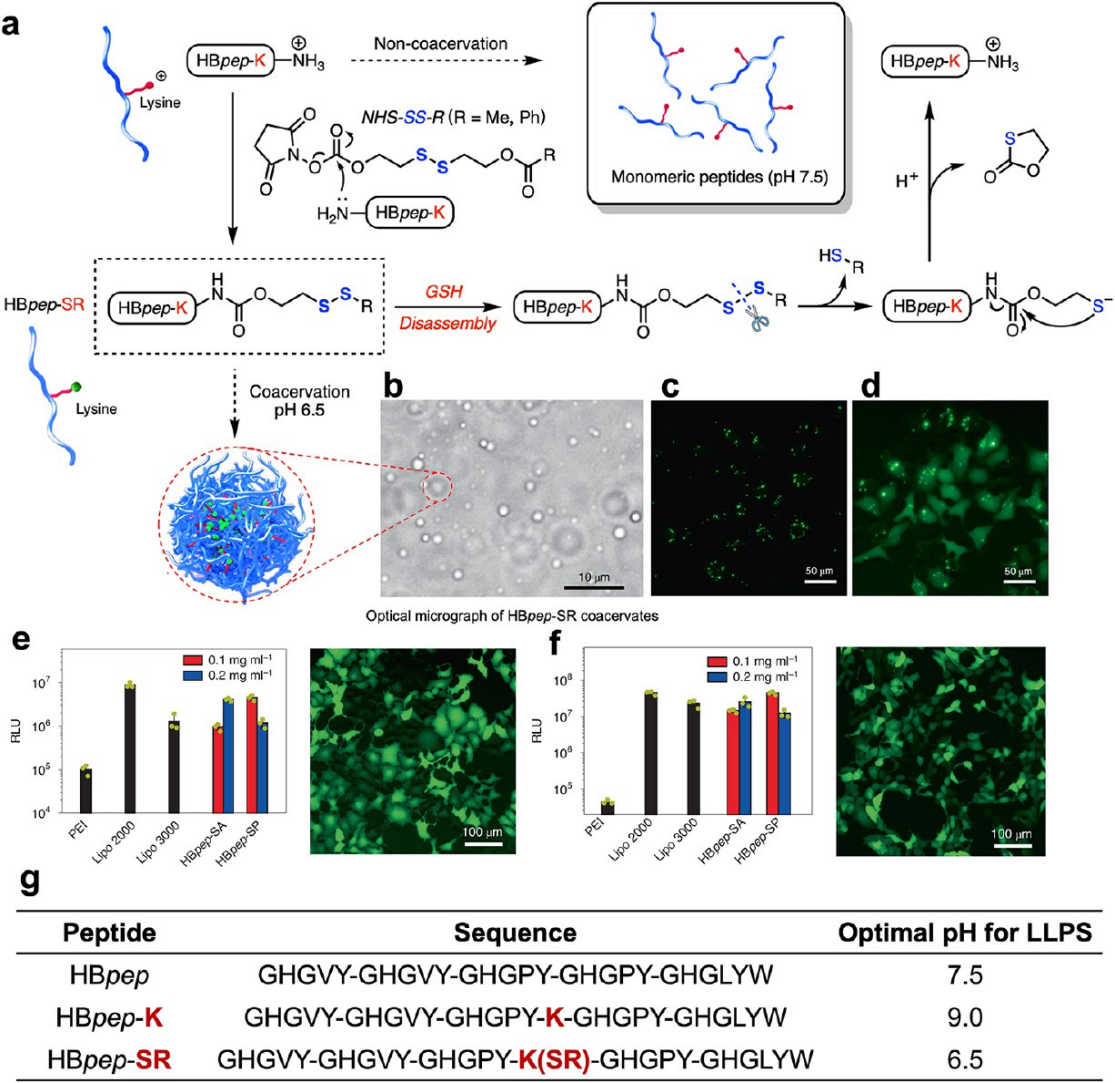
Redox-responsive peptide coacervates HB*pep*-SR for protein delivery and mRNA Transfection. a. HB*pep*-K remains a monomeric peptide at pH 7.5. After the Lys residue in the HB*pep*-K side chain was conjugated with a methyl or phenyl group (HB*pep*-SR, R is methyl, named HB*pep*-SA; R is phenyl named HB*pep*-SP), it undergoes LLPS and forms coacervates. When GSH triggered the reduction of HB*pep*-SR into HB*pep*-K, the coacervates disassembled. b. Optical microscope images of HB*pep*-SR coacervates. c-d. Fluorescent microscope images of EGFP-loaded coacervates-containing HepG2 cells before (c) and after (d) disassembly. e-f. Luciferase-encoding mRNA transfection efficiency of HB*pep*-SA and HB*pep*-SP coacervates compared to common commercial transfection reagents in HepG2 cells (e) and HEK293 cells (f). g. The sequences of peptides HB*pep*, HB*pep*-K, and HB*pep*SR. Reproduced with permission from ref 68. Copyright 2022 Nature Publishing Group.

The phase separation behavior and GSH-triggered cargo release kinetics of HB*pep*-SP coacervates could be modulated by varying the amino acid composition. For instance, the HB*pep*(RPY)-SP variant formed liquid-like coacervate microdroplets (CMs), enabling rapid cellular uptake and payload release. HB*pep*(GP)-SP variant assembled into gel-like CMs, exhibiting slower release kinetics, but was advantageous for sustained nucleic acid delivery. When GP and RPY CMs interacted with giant unilamellar vesicles (GUVs), stiff, gel-like GP CMs induced GUVs’ bilayer bending, while RPY CMs adhered to the membrane, causing deformation and wetting. Some variants could also deliver gene-editing tools. RPY exhibited high efficacy in delivering CRISPR/Cas9 via the ribonucleoprotein (RNP) complex, outperforming even the lipofectamine CRISPRMAX, whereas GP demonstrated lower efficiency (Figures 7 **a-c, g**).^54^ HB*pep*(RP) and HB*pep*(YP) readily formed complex coacervates due to cation-π interactions between Arg and Tyr residues. By tuning the molar ratio of cationic-to-aromatic peptides, the phase behavior and release profiles could be finely optimized. For example, an 8:1 ratio of HB*pep*(RP)-K^SP^ to HB*pep*(YP)-K^SP^ achieved 83.4% EGFP mRNA transfection efficiency in macrophages, highlighting its potential for immune cell engineering (Figures 7 **d, g**).^71^

**Figure 7:**
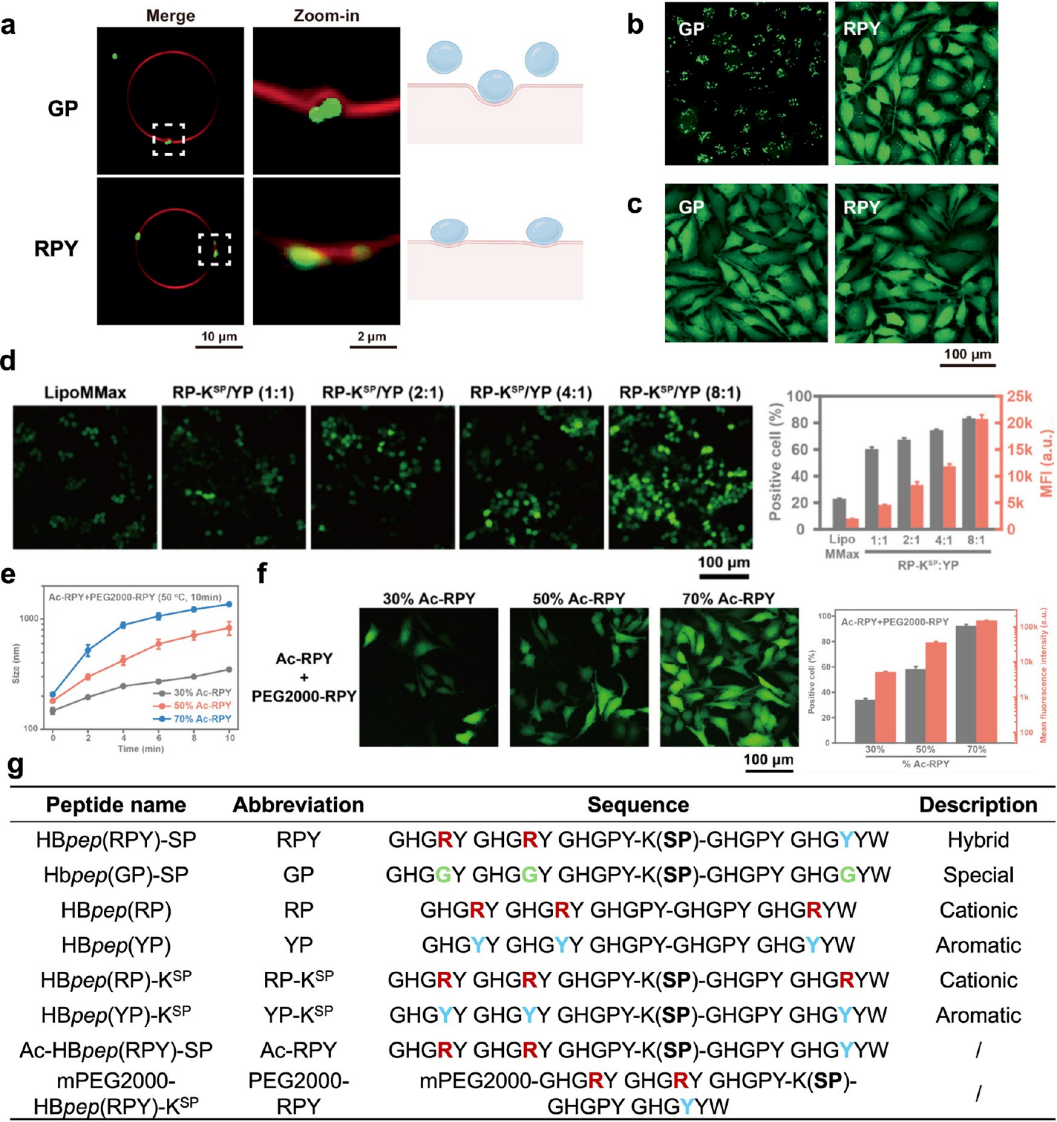
Interaction and internalization of HB*pep*-SP variants coacervates for mRNA transfection. a. GP and RPY CMs interact with GUVs. b. Fluorescence micrographs of HeLa cells treated with EGFP-loaded CMs formed by GP or RPY for 4 h.c. Fluorescence micrographs of HeLa cells treated with EGFP mRNA-loaded GP or RPY CMs for 24 h. Reproduced from ref 54. Available under a CC-BY license 4.0. Copyright 2024 Nature Publishing Group. d. Fluorescence micrographs and FACS measurements of RAW264.7 cells treated with EGFP mRNA-loaded complex coacervates formed by RP-K^SP^/YP with various ratios for 24 h, compared to the commercial reagent Lipofectamine MessengerMax (MMax). Reproduced from ref 71. Copyright 2025 American Chemical Society.e. Average size of Ac-RPY/PEG2000-VPL mixtures at various ratios after incubation at 50 °C for 10 min.f. Fluorescence micrographs and FACS measurements of HeLa cells treated with EGFP mRNA-loaded peptide CNs for 24 h. Reproduced from ref 72. Copyright 2025 American Chemical Society. g. Peptide sequence of HB*pep*-SP variants.

PEGylation enables decreasing and controlling the size of HB*pep* coacervate droplets.^72^ Specifically, poly­(ethylene glycol) (PEG) 2000-conjugated peptides formed stable coacervate nanodroplets (CNs) with diameters ranging from 100 to 200 nm; however, CNs exhibited reduced cargo recruitment efficiency and slower cellular uptake kinetics compared to their micron-sized counterparts. In contrast, peptides conjugated with shorter PEG chains assembled into micrometer-sized coacervate microdroplets (CMs). Distinct cellular internalization pathways were observed between these two systems: CNs predominantly entered cells via clathrin-mediated endocytosis, a mechanism that might reduce macrophage clearance, whereas CMs were primarily internalized by macropinocytosis. To enhance mRNA recruitment and subsequent transfection efficiency, arginine (R) was incorporated into the HB*pep*. Remarkably, a thermally induced phase transition (50 °C, 10 min) triggered the conversion of CNs to CMs, giving a significant increase in cargo-loading capacity, and further boosting mRNA delivery and transfection efficacy, especially at higher RPY ratios (Figures 7 **e-g**).

Besides PEGylation, erythrocyte membranes were also used to stabilize coacervates. Tian et al. developed coacervate droplets formed by sodium hexametaphosphate (SHMP) and mitochondria-targeting antioxidant peptide SS-31 (d-Arg dimethyl Tyr-Lys-Phe-NH_2_), loaded with miRNA-223 (Coac@miR) and coated with erythrocyte membranes (EMCoac@miR) to protect against RNase degradation and enhance circulation stability and targeting.^73^ EMCoac@miR underwent a rapid lysosomal escape via membrane fusion, then GSH-triggered cytosolic release, and simultaneously targeted inflammation (miRNA-223) and oxidative stress (SS-31). Intratracheal (i.t.) injection of Coac@miR directly targeted the lungs, reducing edema and inflammation in acute lung injury (ALI) mice. Intravenous (i.v.) injection of EMCoac@miR achieved 50-times higher lung accumulation than uncoated coacervates.

The study of GHGXY repeats extends beyond peptide sequences, as peptide chirality also plays a critical role in the transmembrane behavior of coacervates. Rudra et al. explored enantiomeric peptide coacervates composed of histidine-rich repeats (GHGXY)_4_ (X = L, V, or P) and their D-amino acid counterparts.^74^ Chirality did not affect LLPS kinetics or droplet characteristics, but hydrophobicity (L > V > P) influences coacervate size. Stronger hydrophobicity resulted in a larger coacervate size and higher encapsulation efficiency. Both L- and D-peptide coacervates entered cells via energy-dependent macropinocytosis, facilitating antigen delivery to lysosomes. D-amino acid coacervates, particularly (GHGVY)_4_, prolong antigen presentation and enhance MHC-I/II pathways, eliciting stronger T cell responses. These coacervates are immunologically inert, making them suitable for vaccine delivery without inducing unwanted immune activation. Enantiomeric coacervates of (GHGVY)_4_ also successfully delivered CRISPR/Cas9 ribonucleoprotein (RNP) complexes in A549 cells, resulting in a corresponding decrease in CD55 expression on the cell surface.

The GSH-responsive dimeric CWWRRRGD coacervates also demonstrated efficient loading and delivery of EGFP-encoding mRNA into multiple cell lines, including HeLa-look, HeLa, A549, NIH-3T3, and HUVEC cells. Successful EGFP expression was observed within 24 h post-transfection, with particularly high transfection efficiency reaching 85.9% in HeLa-look cells.^75^ Dextran nanogel-shielded coacervates (DNSCs) were developed by Dou et al.^76^ The cationic peptide CKKKHHHHKKKC underwent oxidative cross-linking via cysteine residues under ambient conditions to form a redox-sensitive peptide/DNA coacervate core. This core was encapsulated within a shell composed of dextran-poly­(acrylic acid) (Dex-PAA) nanogels cross-linked through disulfide bonds, which was also redox-responsive. Following cellular uptake via endocytosis, DNSCs escaped from lysosomes and were degraded upon exposure to intracellular GSH. The released DNA subsequently entered the nucleus, leading to transgene expression in the cytoplasm.

Beyond GSH reduction, glucose-6-phosphate dehydrogenase (G6PD) can modulate coacervate dynamics via NADPH/NADP^+^ redox cycling. Li et al.^33^ designed DGRR_4_Cs-sCR_4_RGD, a fibrinogen-mimicking, arginine-rich cationic peptide that forms complex coacervates with anionic NADPH. These coacervates encapsulated tissue plasminogen activator (tPA) and shielded it from degradation. It could also target thrombi via RGD-mediated binding, then release tPA sustainably through dual redox triggers, including G6PD-driven NADPH oxidation (coacervate disassembly) and GSH-triggered disulfide cleavage.

#### 3.1.4 Glucose

The first glucose-responsive peptide coacervate was developed by Miserez et al.^77^ using a peptide sequence derived from the Humboldt squid beak protein (DgHBP-2) to form droplets, which encapsulated both insulin and glucose oxidase (GOx) with >95% efficiency. Hyperglycemic conditions (4 mg/mL glucose) resulted in rapid insulin release via pH-induced coacervate dissolution, while normoglycemic levels (1 mg/mL) led to slow release (Figure 8a). Another example is not based on low-molecular-weight coacervates but is also very instructive.^78^ Diethylaminoethyl (DEAE)-dextran/dsDNA coacervate droplets loaded with glucose oxidase (GOx) were coated with erythrocyte membrane fragments, forming the EMCoac protocells. GOx converted glucose to H_2_O_2_, and then hemoglobin (Hb) on the membrane catalyzed H_2_O_2_ and hydroxyurea to produce nitric oxide (NO), resulting in a reduction in blood pressure. Webber et al. established a cationic peptide amphiphile (PA), C_10_-V_2_A_2_R_3_Dap, incorporating a phenylboronic acid (PBA) glucose-binding motif in the Dap position.^79^ Upon binding to high concentrations of glucose, PA–PBA formed a complex with the negatively charged therapeutic protein dasiglucagon, inducing LLPS. These droplets exhibited rapid disassembly in low glucose environments, thereby facilitating the release of dasiglucagon. In a mouse model of insulin-induced hypoglycemia, the droplets improved blood glucose stabilization compared to free dasiglucagon or buffer controls (Figures 8 **b-c**). Webber’s group also provided a deep mechanistic insight into PA–PBA/dasiglucagon LLPS systems, including thermodynamics and structural determinants.^80^

**Figure 8:**
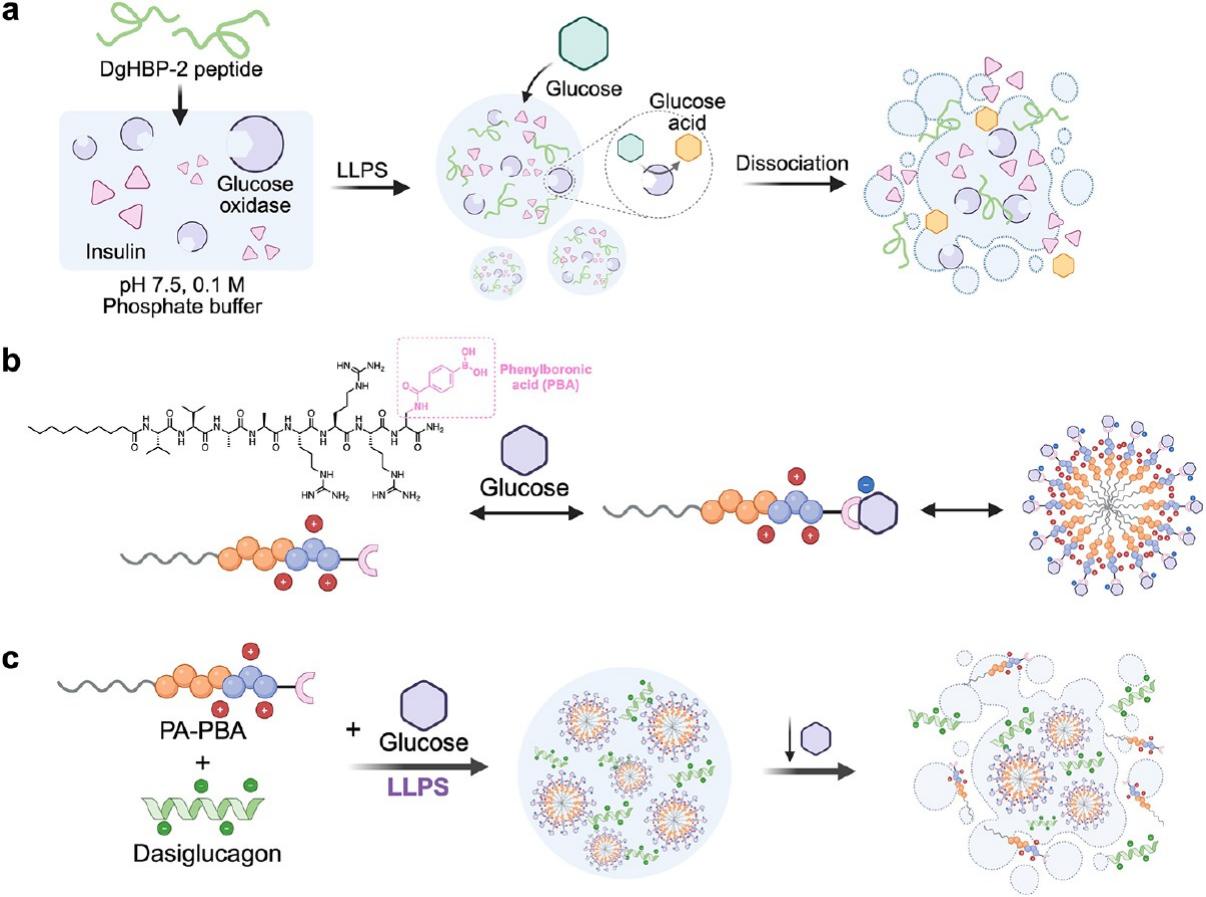
Glucose-responsive coacervates for controlled release. a. Coacervates formed by DgHBP-2 peptides and encapsulation of insulin and glucose oxidase. When the coacervates are exposed to glucose, glucose diffusion into coacervate droplets induces its conversion to gluconic acid, causing localized pH reduction and subsequent droplet dissociation, thereby triggering insulin release. b. Peptide sequence and structure of PA–PBA. When PBA motifs bind to glucose, PA–PBA self-assembles into spherical particles. c. PA–PBA forms a coacervate when it binds to glucose and mixes with dasiglucagon. Reduced glucose concentrations induce droplet dissolution, resulting in dasiglucagon release. Reproduced from ref 79. Copyright 2024 American Chemical Society.

#### 3.1.5 Exogenous Molecules

The small molecules can also achieve responsive release through bioorthogonal reactions. Recently, our lab designed and optimized triphenylphosphine-based compounds capable of forming stable phase-separated coacervates in aqueous solutions.^17^ These microdroplets, termed Staudinger Reaction-Responsive Coacervates (**SR-Coa**), exhibited rapid dissolution upon reaction with azide compounds. The **SR-Coa** system demonstrated remarkable capacity for protein encapsulation, including antibodies, and facilitated their transport across plasma membranes into cellular interiors. Subsequent treatment with ethyl azidoacetate triggered cargo release from condensed puncta to achieve homogeneous cytosolic distribution. Then, they implemented the SR-Coa/ethyl azidoacetate platform to mediate the intracellular delivery of EGFR/antibody complexes, resulting in effective EGFR degradation through the TRIM21-mediated pathway in both in vitro and in vivo. Furthermore, they extended this methodology to target endogenous EZH2 protein, showing the broad applicability of their system. Collectively, this work established a novel strategy for precisely controlling molecular coacervates via intracellular bioorthogonal reactions to achieve cytosolic protein delivery and illustrated its potential for targeted protein degradation through proteasome-dependent pathways (Figure 9).

**Figure 9:**
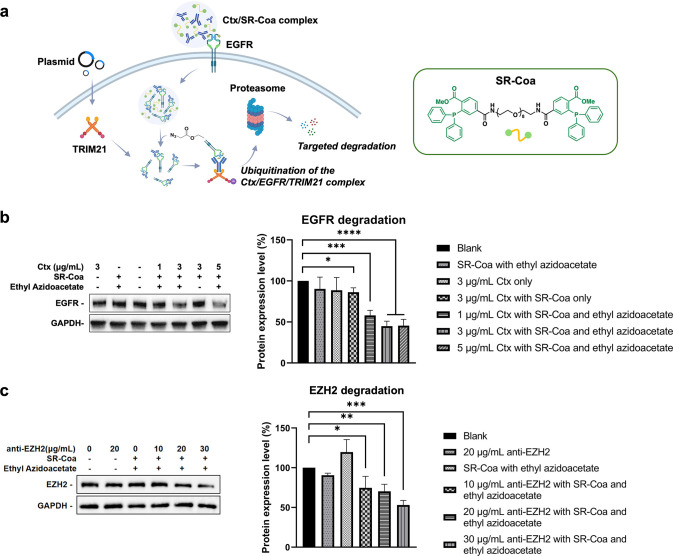
TRIM21-dependent degradation of EGFR and EZH2 mediated by SR-Coa/ethyl azideacetate. a. A schematic illustration showing SR-Coa/ethyl azideacetate-mediated TRIM-AWAY for EGFR degradation. b. Western blotting and statistical analysis of EGFR expression level in different treatment groups. c. Western blotting and statistical analysis of EZH2 expression level in different treatment groups. Reproduced from ref 17. Copyright 2025 American Chemical Society.

#### 3.1.6 Magnetically and Thermally Responsive

Miserez et al. developed an innovative biomimetic peptide-based coacervate system for synergistic hyperthermia and chemotherapy in the treatment of hepatocellular carcinoma.^81^ The coacervates, inspired by histidine-rich proteins found in the Humboldt squid beak, were engineered to coencapsulate doxorubicin (Dox) and magnetic nanoparticles (MNPs). These Dox-loaded Magnetic Coacervates (DMCs) showed excellent biocompatibility and efficient cellular internalization through an endocytosis-independent pathway, coupled with stimuli-responsive Dox release properties upon exposure to an alternating magnetic field (AMF). AMF application induced localized hyperthermia (45 °C), triggering coacervate disassembly and Dox release. In vitro evaluations using hepatocellular carcinoma cells (HepG2) revealed significantly enhanced therapeutic efficacy of the combined thermo-chemotherapeutic approach compared to monotherapies. The DMCs’ unique LLPS characteristics, combined with their colloidal stability and remotely activatable drug release profile, position this platform as a promising theranostic strategy for hepatic malignancies.

#### 3.1.7 Redox and Enzyme Combined

Partial coacervate-based delivery systems exhibited multiple stimuli-responsive properties due to coating with multiresponsive materials. For example, the membrane-bound protocell (PC@MPN) was constructed through the coacervation of oppositely charged oligopeptides (Arg, R10, and Asp, D10) and subsequently coated with a metal-phenolic network (MPN).^82^ Surface modification with folic acid (FA) significantly enhanced the cellular uptake of FA-PC@MPNs in squamous cell carcinoma (SCC7) compared to nontargeted counterparts, PEG–PC@MPNs. When loaded with Dox, FA-PC@MPNs exhibited markedly enhanced cytotoxicity against SCC7 cells, underscoring their potential as a targeted drug delivery platform. The release of therapeutic cargo from PC@MPN nanoparticles was governed by multiple factors. First, the weakly acidic tumor microenvironment (pH 6.5–7.0) or high intracellular GSH levels trigger PC@MPN membrane degradation. Subsequently, MMPs facilitated further decomposition of the membrane components. Lysosomes also contributed to the degradation of the MPN framework, ultimately leading to the release of encapsulated therapeutics.

### 3.2 Cell Surface Engineering

Chimeric antigen receptor (CAR) T-cell therapies have driven advances in immune cell surface engineering, with both natural and synthetic biomaterials investigated to enhance therapeutic effects. However, most fail to fully replicate the dynamic properties of the native extracellular matrix (ECM). Coacervate droplets, beyond their role as potential carriers, exhibit properties that allow them to mimic the ECM. Their capacity to encapsulate and concentrate biomolecules creates a microenvironment that supports biochemical reactions and cellular interactions, mirroring the ECM’s function in structural support and cellular behavior regulation.^83^ Recently, our lab developed an innovative cell surface engineering approach employing phase-separated tripeptides, Fmoc-Lys-Gly-Dopa–OH (KGdelta), which underwent LLPS in aqueous media to form ECM-mimetic coacervates.^84^ Through Fe^3^^+^-mediated coordination, these coacervates spontaneously coated the surfaces of natural killer (NK) cells, forming a functional outer layer capable of incorporating therapeutic monoclonal antibodies, such as trastuzumab (tras). This engineered surface modification conferred upon NK cells the capacity for specific tumor recognition and significantly enhanced antibody-dependent cellular cytotoxicity (ADCC). Our findings established a robust platform for cell surface functionalization and demonstrated its application in arming immune cells with tumor-targeting antibodies (Figure 10).

**Figure 10:**
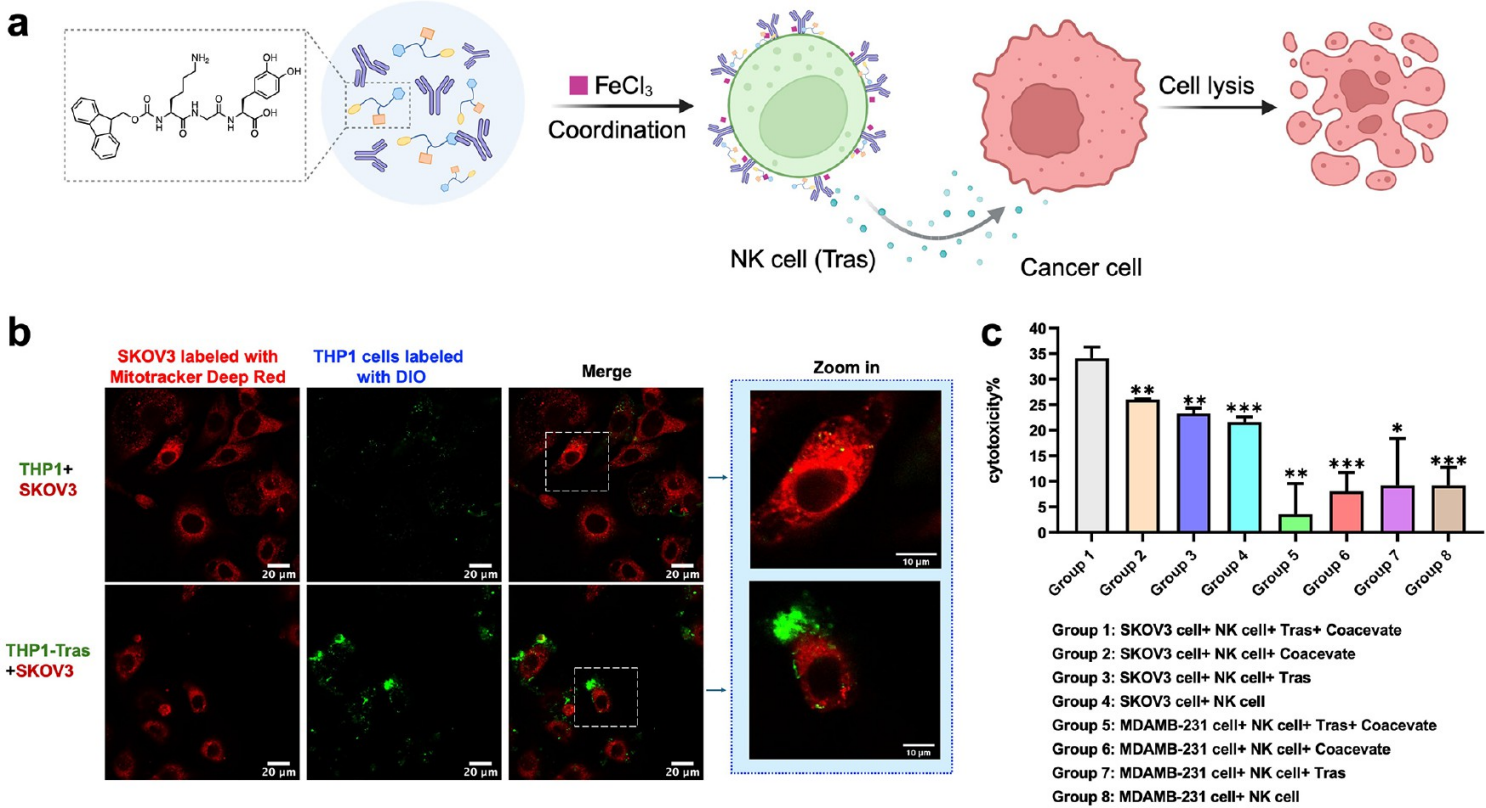
KGdelta-mediated NK Cells surface engineering for cancer cell killing. a. Schematic illustration of KGdelta coating trastuzumab on the NK cell surface with the coordination of FeCl_3_, and then killing the cancer cells. b. Confocal images of the interaction of THP1-Tras with HER2+ SKOV3 cells. c. Cytotoxicity of the cancer cells was measured in different coculture groups. Reproduced with permission from ref 84. Copyright 2024 Wiley-VCH GmbH.

From the perspective of the internalization mechanism of coacervates, some coacervates interact with the cell membrane, which may lead to the formation of new particles with the membrane or potentially cause membrane perforation or leakage. Powerful interactions between coacervates and the cell membrane could also result in higher cytotoxicity; therefore, not all coacervates are suitable as transmembrane delivery carriers. However, the presence of high intracellular toxicity does not necessarily preclude the use of coacervates in therapies. This study provides an excellent solution to address this challenge.

### 3.3 Coacervates in Therapies

The coacervates can serve not only as a delivery vehicle for therapeutic agents but also as a generator of therapeutic agents. Our lab developed spiropyran (**SP**)-based coacervates, SP-(PEG)_8_-SP (**SP-C**), as dual-wavelength-switchable generators of reactive oxygen species (ROS) for photodynamic therapy.^18^ UV light (365 nm) triggered **SP-C** isomerization into fluorescent merocyanine (**MC-C**, MC-(PEG)_8_-MC), while visible light (565 nm) reversed the process and simultaneously induced ROS generation. The coacervate state enhanced the efficiency of ROS generation compared to dispersed **MC** molecules. Intravenously injected **SP-C** accumulated in the tumor site 3 h after administration and reached the peak at 6 h before gradually diminishing. Dual-wavelength illumination (404/565 nm) triggered localized ROS production, significantly suppressing breast tumor growth without systemic toxicity (Figure 11). Jiang et al. engineered a multiphase core–shell coacervate (CSC) system through the assembly of dsDNA and somatostatin (SST), a 14-amino acid cyclic peptide, which conferred tumor-targeting capability via binding to somatostatin receptors (SSTRs) on tumor cell surfaces.^85^ The CSC facilitated the incorporation of the G4 quadruplex-hemin complex, which then recruited the photosensitizer (PS) tetrakis­(4-carboxyphenyl) porphyrin (TCPP), resulting in the formation of a CSC-G4/hemin-TCPP (CSC-GHT) for enhanced photodynamic therapy (PDT). Notably, the CSC-GHT system exhibited about a 2.4-fold increase in singlet oxygen (^1^O_2_) generation compared to free TCPP under 660 nm laser irradiation, substantially enhancing PDT efficacy. Unlike conventional PDT systems, where PS aggregation often resulted in quenching and a reduction in reactive oxygen species production, the coacervate effectively concentrated TCPP in a nonaggregated state, thereby mitigating aggregation-caused quenching and enabling sustained molecular exchange with the microenvironment, rather than a burst release. This synergistic mechanism enabled the effective inhibition of cellular migration in vitro and the suppression of tumor growth in vivo.

**Figure 11:**
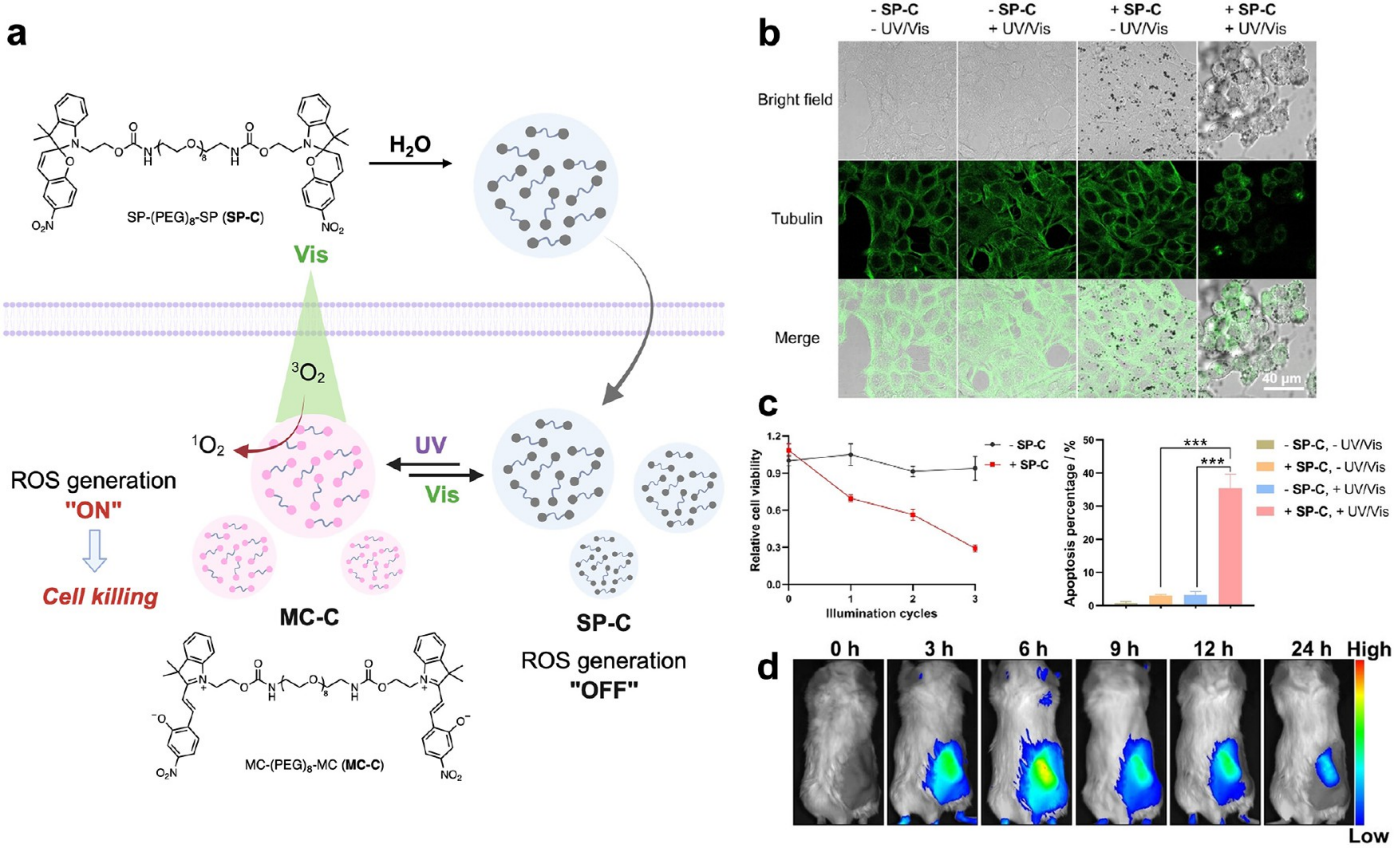
Light-induced transformation and cytotoxicity of spiropyran-based coacervates in HeLa cells. a. Schematic illustration of the light-induced transformation between SP-C and MC-C. Under 365 nm illumination, SP-C converts to MC-C. When exposed to 565 nm light, MC-C then generates ROS and induces cell death. b. Confocal images of photoinduced morphology change of HeLa cells. c. CCK-8 and cell apoptosis assay of photoinduced HeLa cell killing. d. Whole-body fluorescence imaging showing the accumulation of the spiropyran coacervates at the tumor site. Reproduced with permission from ref 18. Copyright 2025 Wiley-VCH GmbH.

These studies ingeniously exploit the property of coacervates to accelerate localized reaction kinetics and successfully translate this concept into in vivo applications. In addition, our system operated without relying on coacervates for therapeutic agent delivery, thereby significantly simplifying the design and experimental workflow. Jiang’s system utilized inherent properties of the coacervate to overcome the disadvantages of the compound, such as aggregation. These two approaches provide a novel paradigm for translating coacervate-based systems into clinical therapeutics.

### 3.4 Others

#### 3.4.1 Inhibition of Cell Proliferation

Uesugi et al. screened 843 types of self-assembling small molecules and ultimately discovered that huezole can selectively bind to tubulin and form droplets.^19^ This small molecule entered cells and formed droplets that could capture tubulin, thereby preventing its normal polymerization and affecting cell division. In HeLa cells, treatment with huezole resulted in significant inhibition of cell proliferation, with an IC_50_ value of 4.4 mM. As the concentration of huezole increased, the proportion of cells in the G2/M phase of the cell cycle rose, indicating its impact on the cell cycle. Although no specific therapeutic scenarios are provided, this also offers ideas for utilizing LWMC-based condensates as tools for disease treatment.

#### 3.4.2 Adhesive

GK-16* (GY*KGKY*Y*GKGKKY*Y*Y*K, Y* represents Dopa), a peptide from the adhesive protein Mfp-5, forms coacervates in seawater, with Dopa- and Gly-mediated hydrogen bonding being crucial for this process. The properties of these coacervates could be adjusted by modifying the pH and salt concentration, affecting the strength of electrostatic and hydrogen bond interactions. Additionally, GK-16* coacervates exhibited a pH-induced liquid-to-gel transition, enabling their use for underwater adhesive delivery and curing.^86^

## 4 Conclusion and Outlook

In recent years, LMWC-based coacervates primarily composed of short peptides and small molecules have made significant advancements in drug delivery. The phase separation behavior of short peptides can be fine-tuned through the side chain groups of amino acids. Varying the aromaticity of amino acids allows for adjustments in overall aromaticity to regulate the molecule’s interacting force. Additionally, the distinct isoelectric points of amino acids enable regulation of phase behavior through pH adjustments. Modifications to the peptide backbone or side chains can also influence phase separation, allowing for coacervation or disassembly by removing modifying groups.^68,87^ Small molecules exhibit similar properties, where phase behavior and functionality can be modulated by altering sticker groups. Furthermore, spacers can be adjusted; for example, disulfide bonds can be introduced for redox regulation, and the length of the PEG linker can be varied to modify hydrophilicity. Besides, some cationic cell-penetrating peptides (CPPs) possibly contribute to the formation of coacervates by interacting with negatively charged molecules, particularly nucleic acids and proteins. CPPs may facilitate the internalization of these coacervates, leading to improved delivery efficiency. The release of cargo from coacervates can also be modulated by the presence of CPPs, which may alter the coacervate’s stability in response to cellular conditions.

A critical question in exploring coacervate-mediated transmembrane delivery is how coacervates enter cells. Although current research on the transmembrane mechanisms of LMWC-based coacervates is limited, discernible patterns can still be identified. Partial view suggests that the internalization pathway of LMWC-based coacervates exhibits a unique hybrid mechanism, combining features of both phagocytosis and macropinocytosis. Additionally, this internalization pathway is cholesterol-dependent and involves lipid rafts. Cholesterol influences the recognition and binding of coacervates to the cell membrane by modulating its rigidity. An increased cholesterol content in the cell membrane enhances its bending rigidity, thereby facilitating the binding of coacervates to the membrane and strengthening their interactions, ultimately improving internalization efficiency. Furthermore, the interaction between amino acid side chains and the β-hydroxyl group of cholesterol may enhance coacervate-membrane binding and promote the cellular uptake of simple coacervates.^52^ Moreover, stronger membrane adhesion through electrostatic interactions with positively charged residues can accelerate uptake. 3D confocal imaging provides conclusive evidence that LMWC-based coacervates enter cells as micron-scale droplets, not as monomers or small aggregates.^16–18,68^ The cytoplasmic presence of micrometer-sized, spherical droplets is observed. Monomers would diffuse, and small aggregates would form irregular shapes, neither of which is consistent with the observed morphology.

The internalization mechanisms of coacervates are complex and diverse, which might be intrinsically linked to their physicochemical properties. As dynamic and unstable liquid droplets with heterogeneous sizes, coacervates exhibit diverse cellular internalization pathways. Larger coacervates tend to favor macropinocytosis, while smaller coacervates, resembling nanoparticles, prefer endocytosis.^44^ Notably, larger coacervate droplets resist complete engulfment due to the accumulation of membrane tension during the wrapping process.^54^ Furthermore, the fluidity of coacervates, which varies between different systems, might influence their interfacial interactions with cell membranes. Coacervates with higher fluidity demonstrate stronger membrane engagement and faster internalization, while less fluid counterparts internalize more slowly. Lastly, the differential surface charges of various coacervates contribute to their distinct interactions with cell membranes.

Currently, coacervates used for delivery primarily rely on intracellular endogenous stimuli, including GSH, various enzymes, and glucose. Tumor cells with high GSH levels facilitate the disassembly of coacervates in the tumor microenvironment, enabling effective release. Enzyme-responsive coacervates offer notable advantages for targeted delivery. Glucose-responsive systems are particularly intriguing, as they leverage fluctuations in intracellular glucose concentrations to regulate coacervate self-assembly and disassembly, promoting drug release in target cells and achieving sustained release of bioactive molecules, such as insulin, in response to physiological conditions. Additionally, exogenous stimuli such as light and small molecules provide further control over coacervate-based delivery. Light irradiation provides spatiotemporal precision for therapeutic release, while small molecules can diffuse into cells, inducing rapid coacervate disassembly and cargo release. While each approach possesses distinct advantages, redox (GSH)-responsive systems are the most promising strategy for broad-spectrum therapeutic applications, particularly in oncology. This approach exploits the characteristically elevated GSH levels within the cancer cell cytosol, which provides a universal and naturally targeted mechanism for triggered intracellular release. Furthermore, GSH-responsive coacervates achieve superior delivery efficacy and remarkable versatility, enabling the efficient cytosolic transport of a diverse array of macromolecular cargo, from proteins and mRNAs to CRISPR/Cas9 gene-editing tools, and even outperforming conventional commercial reagents.

Despite the high delivery and therapeutic efficiency of these LMWC-based coacervates, precise targeting remains a key challenge. Enzyme-responsive coacervates can achieve localized formation or release by recognizing tumor or inflamed tissue-specific enzymes; however, off-target effects, such as nonspecific enzymatic degradation or cross-reactivity with structurally similar substrates, pose limitations. Moreover, enzyme-triggered kinetics are sometimes slow. To address these challenges, the development of multistimuli-responsive coacervates that integrate complementary triggers could enhance release speed, sensitivity, and accuracy.

On the other hand, not all LMWC-based coacervates are suitable for transmembrane delivery due to their varying levels of cytotoxicity. It is hypothesized that the cytotoxicity induced by different coacervates may stem from their distinct interaction mechanisms with cellular membranes. The proposed hypotheses are as follows: First, some coacervates may interact with lipids, promoting lipid rearrangement in the cell membrane, which could alter membrane fluidity and structural integrity, thereby triggering cellular stress responses and inflammatory reactions that exacerbate cell damage. Second, excessively large coacervate droplets may exert mechanical compression on membranes, creating abnormal tension that disrupts normal cellular functions. Third, certain coacervates may specifically bind to membrane receptors or proteins, leading to aberrant activation or inhibition of associated signaling pathways. The reasons for the cytotoxicity induced by coacervates are worth exploring.

Although numerous LMWC-based coacervates capable of transmembrane delivery have been reported, our understanding of these systems remains limited. A major obstacle is the lack of diverse models to guide the discovery and design of such molecules. Current approaches primarily focus on identifying highly repetitive sequences within proteins or utilizing the ″sticker-spacer″ model for designing LMWC-based phase-separating molecules. Machine learning has significantly improved the prediction of phase-separating sequences and optimized design parameters.^88^ Nonetheless, assessing the suitability of computationally predicted coacervates for transmembrane delivery remains challenging due to the out-of-equilibrium and highly crowded nature of the cellular context.

Finally, a key challenge in understanding coacervate-based protocells in the origins of life is elucidating their transition toward higher-order complexity and life-like functionalities.^89^ Membraneless protocells inherently suffer from instability issues, including susceptibility to Ostwald ripening, coalescence, surface spreading, and dissolution. Surface modifications using PEG, lipids, erythrocyte membranes, and other biomaterials can enhance the structural stability of coacervates. Although significant progress has been made in studying the stability and transmembrane delivery mechanisms of coacervate protocells, several fundamental questions remain unresolved: (1) How can we precisely regulate coacervate membrane properties (thickness, permeability, selectivity) and achieve dynamic tunability to better mimic biological membrane functionality? (2) How can we balance the inherent tension between structural stability and efficient transmembrane delivery? In complex intracellular environments, how can coacervates maintain environmental adaptability while facilitating selective molecular exchange? (3) How can we rigorously verify that the observed stabilization and delivery mechanisms in coacervates truly reflect plausible pathways for early life emergence? (4) Does increasing structural complexity through engineering modifications compromise the native advantages of coacervates, such as operational simplicity, avoidance of organic solvents, and high cargo recruitment efficiency? If so, how do they fundamentally differ from conventional vehicles beyond mere size differences? These unresolved questions highlight a critical research frontier where multidisciplinary approaches must converge to engineer functionally advanced, life-like protocells based on LLPS principles.
